# Probiotics in Pet Food: A Decade of Research, Patents, and Market Trends

**DOI:** 10.3390/foods14193307

**Published:** 2025-09-24

**Authors:** Phatthranit Klinmalai, Pitiya Kamonpatana, Janenutch Sodsai, Atcharawan Srisa, Khwanchat Promhuad, Yeyen Laorenza, Attawit Kovitvadhi, Sathita Areerat, Anusorn Seubsai, Massalin Nakphaichit, Nathdanai Harnkarnsujarit

**Affiliations:** 1 Faculty of Agro-Industry, Chiang Mai University, Samut Sakhon 74000, Thailand; phatthranit.k@cmu.ac.th; 2 Department of Food Science and Technology, Faculty of Agro-Industry, Kasetsart University, Bangkok 10900, Thailand; fagipyk@ku.ac.th; 3 Department of Packaging and Materials Technology, Faculty of Agro-Industry, Kasetsart University, Bangkok 10900, Thailand; janenutch.s@ku.ac.th (J.S.); atcharawan.sri@ku.th (A.S.); khwanchatpromhuad@gmail.com (K.P.); yeyen.la@ku.th (Y.L.); 4 KU Vet Innova Nutricare Co., Ltd., Kasetsart University, Bangkok 10900, Thailand; attawitthai@gmail.com; 5 Department of Physiology, Faculty of Veterinary Medicine, Kasetsart University, Bangkok 10900, Thailand; sathitameen@gmail.com; 6 Department of Chemical Engineering, Faculty of Engineering, Kasetsart University, Bangkok 10900, Thailand; fengasn@ku.ac.th; 7 Department of Biotechnology, Faculty of Agro-Industry, Kasetsart University, Bangkok 10900, Thailand; fagimln@ku.ac.th; 8 Center of Excellence for Microbiota Innovation, Faculty of Agro-Industry, Kasetsart University, Bangkok 10900, Thailand

**Keywords:** probiotic, pet food, gut microbiota, microencapsulation, synbiotic, canine nutrition

## Abstract

Increasing interest in functional nutrition has driven the incorporation of probiotics into pet food formulations to enhance digestive health, immune response, and overall well-being in companion animals. This systematic review examines scientific publications, patents, and market developments related to probiotic-enriched pet food from 2014 to 2024. We evaluate major probiotic taxa—including *Lactobacillus* spp., Bifidobacterium spp., *Bacillus* spp., and *Saccharomyces cerevisiae*—based on their resilience during processing, gastrointestinal survival, and documented health benefits. Delivery technologies such as microencapsulation, coating matrices, and post-processing supplementation are analyzed for their effectiveness in preserving probiotic viability within dry and wet food matrices. Patent landscape analysis highlights innovation trends in strain selection, formulation design, and processing methods. In vivo and in vitro studies demonstrate that probiotic supplementation modulates gut microbiota composition, improves fecal parameters, enhances immune markers, and promotes nutrient absorption in both canine and feline models. Market data reveal rapid expansion of commercial probiotic pet food products, yet scientific research remains limited compared to human nutrition. Overall, the findings indicate that while probiotics hold clear potential to improve gastrointestinal health and immunity in pets, evidence remains fragmented, particularly for cats and long-term outcomes. Bridging the gap between industrial innovation and controlled clinical validation will be essential for developing next-generation probiotic pet foods.

## 1. Introduction

The growing market demand for probiotic-enriched pet food reflects pet owners’ increasing pursuit of health-promoting functions such as improved digestion, immune support, and overall well-being of companion animals [1,2,3]. Probiotics, defined as live microorganisms that confer benefits on the host when administered in adequate amounts [2], are already well established in human nutrition and are now increasingly applied in dog and cat diets [3,4,5]. However, current industrial adoption faces major bottlenecks, particularly the low survival rate of probiotics during high-temperature extrusion, storage, and gastrointestinal transit, which reduces their efficacy and challenges product credibility [6,7,8]. Addressing these constraints requires integrated strategies that connect strain selection with technological protection systems and practical food processing applications. Therefore, the core objective of this systematic review is to provide guidance on probiotic strain selection, technological applications to enhance viability, and industrial transformation to align consumer demand with scientifically validated, functional pet foods. Technological advancements like microencapsulation, hydrogel matrix drying, and post-extrusion coating have been developed to boost the stability and viability of probiotics in dry kibble, wet food, snacks, and treats [9,10]. Despite robust market growth, evidenced by a sharp rise in probiotic pet food product launches since 2018, academic publications remain comparatively limited [1]. This disparity suggests an industry focus on innovation through proprietary technologies and patents rather than extensive public dissemination of research findings. Recent patents highlight novel processing techniques, strain selection strategies, and synergistic formulations aimed at maximizing probiotic efficacy in pet foods [3].

In addition to commercial interests, scientific investigations have confirmed the functional benefits of probiotics in companion animals, including modulating gut microbiota, enhancing short-chain fatty acid production, improving fecal consistency, and strengthening immune responses [6,11]. However, challenges persist regarding probiotic strain specificity, optimal delivery matrices, and maintaining viable cell counts throughout processing and storage [7,9]. Furthermore, opportunities exist for expanding probiotic applications tailored to specific life stages, such as puppies, kittens, and senior pets, where gastrointestinal and immune health support is critically important [2,8]. This review aims to provide a comprehensive analysis of the trends, applications, challenges, and innovations associated with probiotic use in pet foods. It examines probiotic strain diversity, delivery technologies, health benefits, patent activity, and future directions, offering insights for researchers, industry stakeholders, and veterinary professionals involved in developing next-generation functional pet foods.

## 2. Trends in Probiotics in Pet Food

**Figure 1:**
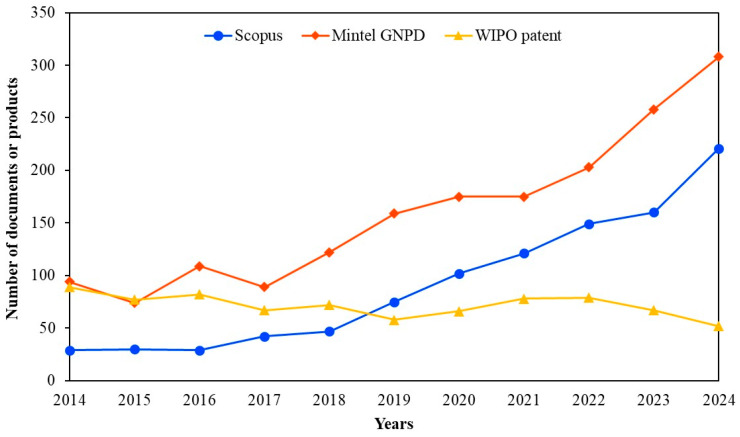
Annual trends in the number of records retrieved from Scopus (for article publications), the World Intellectual Property Organization (WIPO) (for patents), and the Mintel Global New Products Database (GNPD) (for product launches) between January 2014 and December 2024. For the Scopus data, the search was conducted using the keywords: “pet,” “dog,” “cat,” “canine,” or “feline” in combination with “probiotics.” For WIPO and Mintel GNPD, the keywords used were: “pet food,” “dog food,” or “cat food” in combination with “probiotics”.

Pet owners are becoming more interested in probiotics for functional diets which have led to a rise in the demand for pet health products in recent years. The incorporation of probiotics into pet food has the potential to enhance nutrient digestibility, improve the quality of feces and support immune health in dogs and cats. Figure 1 shows the comparative annual worldwide trends in probiotic pet food collected from Scopus (for academic publications), the World Intellectual Property Organization (WIPO) (for patents), and the Mintel Global New Products Database (GNPD) (for global product launches). Although academic publications on probiotics in pet food were initially limited, the number of articles indexed in Scopus has shown a clear upward trend since 2018, with a notable acceleration after 2020. This growing academic attention aligns with increasing industrial innovation and consumer demand for probiotic-enriched pet food products. The industry seems to be investing in novel technologies such as new probiotic strains, encapsulation methods or processing techniques to enhance product value and maintain competitive advantage. Filing patents allows enterprises to protect their innovations and obtain intellectual property rights which are crucial for distinctive items in a quickly growing market. The Mintel GNPD shows the strongest trend with probiotic pet food launches rising sharply from 2018 and reaching over 100 new products through 2024. This growth indicates a strong and growing consumer demand for functional pet food products enriched with probiotics. Many new products may use similar probiotic strains or technologies that are not different enough to qualify for a patent. In some cases, companies may prefer to launch products quickly rather than spend time and money applying for a patent.

**Figure 2:**
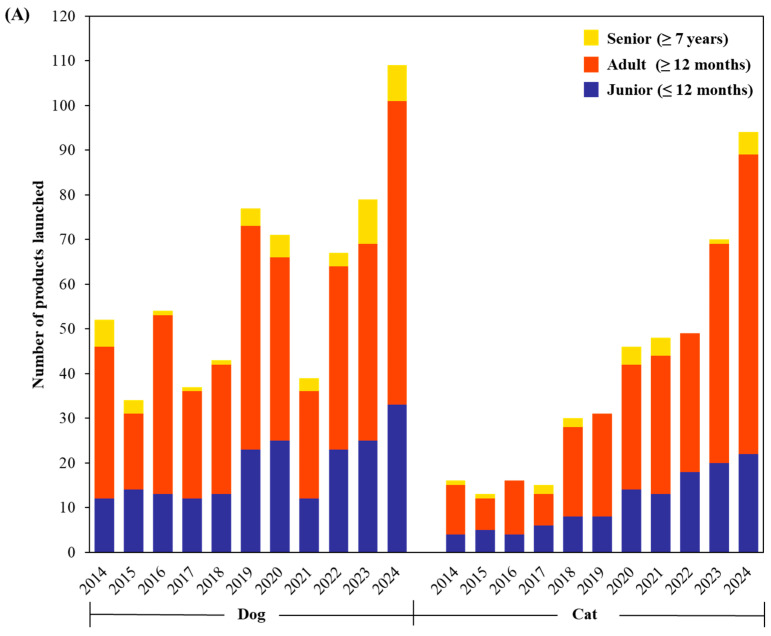

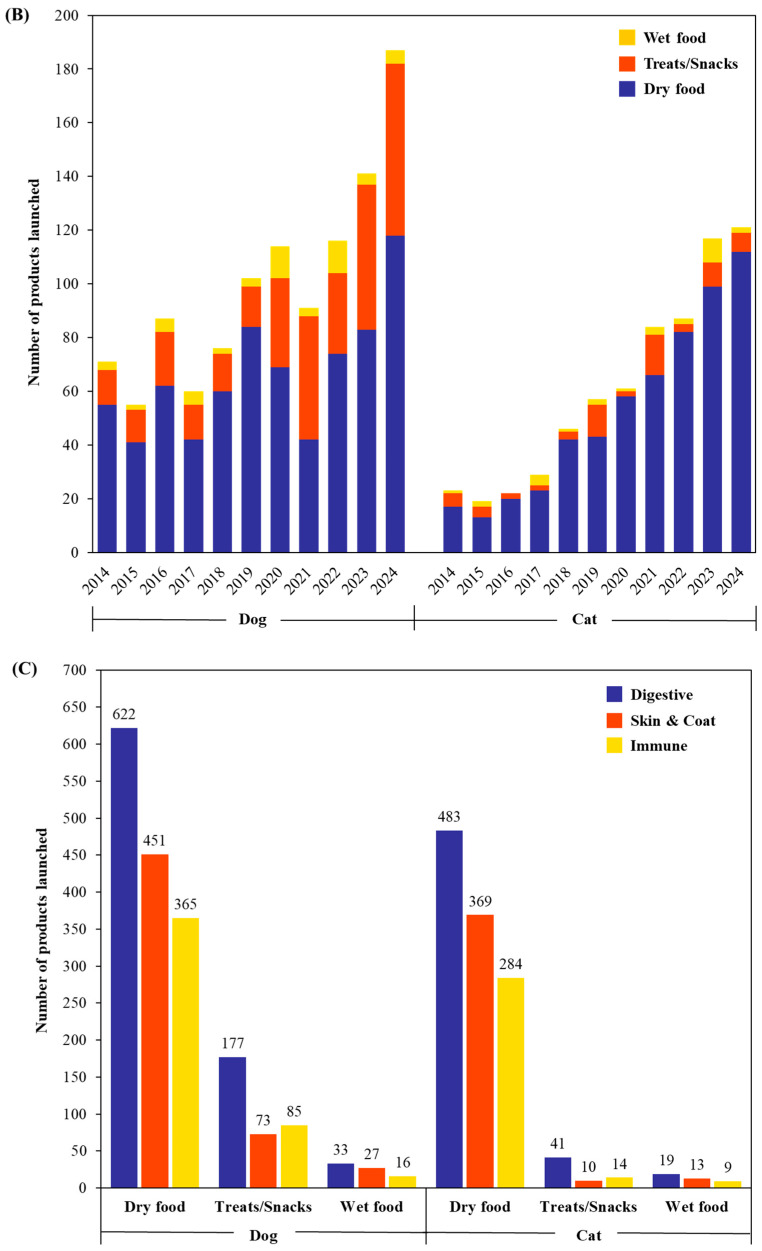
Annual trends in probiotic pet food product launches insights from Mintel GNPD (January 2014 to December 2024) categorized by (**A**) ages (senior, adult, and junior), (**B**) food type (wet food, treats/snacks, and dry food) and (**C**) functional claims (digestive, skin and coat, and immune) for dogs and cats.

Probiotics are increasingly incorporated as functional ingredients in premium pet foods, snacks, and treats. This trend reflects the growing interest of pet owners in health, wellness, and functional nutrition. Figure 2A shows annual trends in probiotic pet food launches between 2014 and 2024, classified by life stage for both dogs and cats based on Mintel GNPD data. The “Junior” category refers to pets aged 0–12 months, “Adult” to those over 1 year, and “Senior” to those over 7 years [12]. Across the decade, probiotic products targeting adult dogs and cats represent the largest segment. The number of adult-targeted launches increased markedly after 2018, peaking by 2024, especially for dogs. This growth corresponds with heightened consumer awareness of gut health, immune function, and overall wellness maintenance in mature pets. In contrast, products formulated for juniors (puppies and kittens) represent a smaller but steadily growing segment, with modest growth observed from 2020 onwards. The lower representation in this category may be due to the short duration of this life stage and relatively lower consumer demand. Although the senior segment is still smaller than the adult category, its steady growth reflects increasing recognition of the unique nutritional and health needs of aging dogs. At the same time, the limited number of corresponding products may be due to formulation challenges, fewer feline-specific studies, and slower market adoption, highlighting a gap between research efforts and commercial outcomes.

Figure 2B illustrates probiotic pet food launches segmented by food type. Among the three categories, dry food consistently accounts for the majority of probiotic product launches across all years. Despite challenges in maintaining probiotic viability during commercial extrusion, dry food remains the most widely used format due to its convenience, long shelf life, and cost-effectiveness. Treats and snacks have shown a sharp increase since 2020, reflecting growing demand for functional treats that offer health benefits in flexible and chewable formats. In contrast, wet food remains the least represented format, despite offering palatability benefits and being suitable for specific dietary needs. The limited use of probiotics in wet food is likely due to greater difficulties in maintaining viability during processing and storage. These data underscore the market’s strong inclination toward dog-focused probiotic innovations, particularly in dry formats. Although cat products are underrepresented, this gap highlights opportunities for growth in feline-specific probiotic offerings.

**Figure 3:**
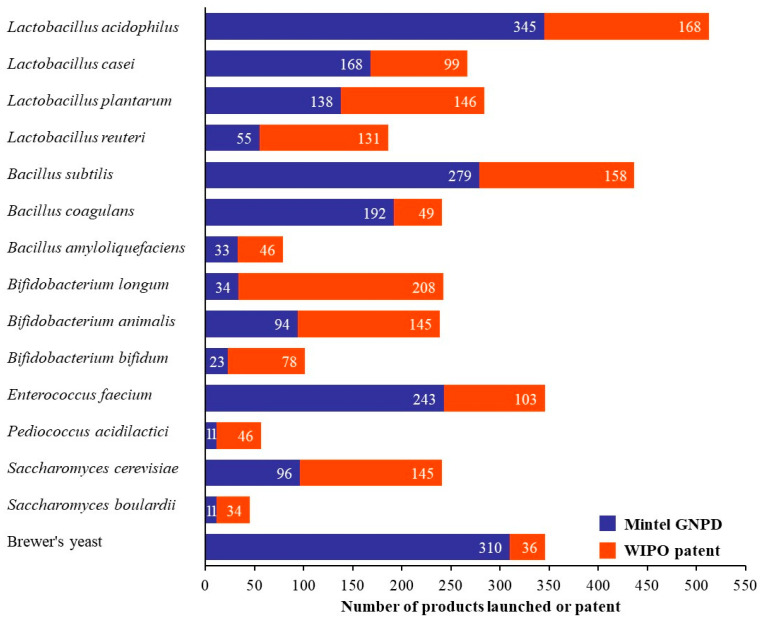
Probiotic strains used in commercial pet food products and related patents from Jan 2014 to Dec 2024 based on data from Mintel GNPD and WIPO patents.

Figure 3 presents a comparative analysis of probiotic strains used in commercial pet food products based on data from Mintel GNPD and their intellectual property trends from the WIPO patent database. Probiotic lactic acid bacteria including *Bifidobacterium* and *Lactobacillus*, are widely used in food and fermented dairy products because they are generally recognized as safe (GRAS) [13]. Among *Lactobacillus* strains, *Lactobacillus acidophilus* emerges as the most dominant strain in commercial products and patents, followed closely by *Lacticaseibacillus casei* and *Lactiplantibacillus plantarum*. *L. acidophilus* has been reported as a potential probiotic in pet food for enhancing oral health in dogs [3] and effectively improving intestinal health in pets [6]. Although *L. plantarum* and *L. casei* have been studied for their potential to enhance immune function and intestinal health in dogs [14], their use in commercial pet food remains less common compared to *L. acidophilus*. This may be attributed to their traditional application in fermented foods for human consumption such as yogurt, kimchi, and sauces and others [15]. In contrast, *L. acidophilus* has gained broader acceptance in the pet food industry due to its stronger association with gastrointestinal health and its relatively higher tolerance to processing conditions and gastrointestinal transit in companion animals [8]. In contrast, *Limosilactobacillus reuteri* shows moderate product launches but surprisingly high patents due to requiring new and innovative strategies in commercial applications [16]. *Bifidobacterium longum* and *Bifidobacterium animalis* have more patents but limited product launches, indicating their perceived value in innovation and potential future applications. *B. bifidum* remains limited due to its low tolerance to environmental conditions during processing and in the gastrointestinal tract [17]. This results in lower survival rates and the use of this strain in functional pet food. *Bacillus* strains, particularly *Bacillus subtilis* and *Bacillus coagulans*, are attractive for use in commercial pet food launches and patents, not only in their health-promoting functions but also in their robustness, thermostability and resistance to acidic pH [18]. These attributes are essential for surviving heat-treated food processes and the gastrointestinal tract. *Bacillus* strains are well-suited for use in heat-processed pet foods such as dry kibble or baked treats [19]. In contrast, more sensitive strains like *Bifidobacterium* or *Lactobacillus* often degrade during these processes, making *Bacillus* a more viable probiotic candidate for dry pet food formulations [18]. *Enterococcus faecium* is a prevalent probiotic strain in 243 commercial pet food products and 103 patents (Figure 3). It is commonly found in the gastrointestinal tract of both dogs and cats. It can survive for months in the environment and tolerance to a range of adverse conditions including acidic pH and bile salts which enhances its viability in processed pet food matrices [8]. *Pediococcus acidilactici* has emerged as a novel and promising probiotic strain in the companion animal sector. Although it appears in only 11 commercial pet food products and 46 patents (Figure 3) reflect growing research interest. This strain is valued for its ability to survive and exhibit bacteriocins in the host gastrointestinal tract and effectively inhibiting bacterial pathogens [20]. *Saccharomyces cerevisiae* and *Saccharomyces boulardii* are both yeast-based probiotics. These strains are particularly relevant in digestive and immune-support formulations and offer alternative probiotic mechanisms compared to bacterial strains [21]. *Saccharomyces cerevisiae* is commonly referred to as brewer’s yeast or deactivated brewer’s yeast in probiotic pet food included 406 commercial products and only 181 related patents. It has been shown to offer nutritional versatility in formulating alternatives to rendered protein meals for pet foods and treats [22] and may help to reduce gut permeability in adult dogs [23]. Overall, the trends analyzed in this study reflect both current consumer and industry preferences, while also forecast the next generation of probiotic innovations in the pet food sector.

While market expansion underscores the rising demand for probiotic pet food, scientific validation remains essential to support product credibility. Therefore, the following section reviews research findings on probiotics in companion animals, emphasizing strain-specific effects and their health outcomes.

## 3. Research and Applications of Probiotics in Pet Food

The incorporation of probiotics into pet food has been widely explored, with research emphasizing microbial viability, health outcomes, and formulation strategies to ensure functional efficacy. Probiotics used in pet food between 2014 and 2025 are reviewed, as shown in Table 1. A broad range of probiotic strains—including *L. plantarum* CIDCA 83114, *L. acidophilus* DSM13241, *L. johnsonii* CPN23, *L. reuteri* AI, *L. murinus* LbP2, *B. longum* KACC 91563, *B. subtilis* C-3102, *B. amyloliquefaciens* CECT 5940, *E. faecium* NCIMB10415, SF68, and CECT 4515, as well as multispecies blends containing *Streptococcus thermophilus*, *L. casei*, *L. paracasei*, *L. delbrueckii*, *B. breve*, *B. infantis*, *B. bifidum*, and *Weissella cibaria* JW15—have been evaluated across various delivery matrices, including biscuits, extruded kibble, cheese, and dietary supplements [2,6,7,9,24,25,26,27,28,29,30,31,32,33].

### 3.1. Gut Microbiota Modulation

Studies consistently report improvements in gut microbiota composition, notably increases in *Lactobacillus* and *Bifidobacterium* populations and reductions in pathogenic organisms like *Clostridium perfringens* and *Enterobacteriaceae* [2,25,30]. Processing methods such as protective coating [9] and supplementation with viable cultures post-extrusion or through fermented matrices have proven effective in maintaining probiotic stability. Kefir, a fermented milk containing the living probiotic *Lactobacillus kefiri* has successfully modified gut microbiota in dogs [34,35]. Kefir consumption decreased *Clostridiaceae*, *Fusobacteriaceae*, and *Ruminococcaceae* in dogs while increasing the population of lactic acid bacteria and the lactic acid bacteria:Enterobacteriaceae ratio. This mechanism is related to the competition between probiotic species and these microbiotas [35].

### 3.2. Clinical Applications in Companion Animals

Lactobacillus species are the most studied and used probiotics in veterinary medicine due to their ability to improve fecal parameters, resulting in softer and less watery stools [36,37,38,39]. Bruni et al. (2020) [36] reported that *L. acidophilus* D2/CSL (CECT 4529) supplementation could prevent being overweight by maintaining an ideal body condition score (BCS) with thinner skin and improved fecal parameters compared to dogs on a control diet. Reduced skin thickness at the neck and thorax is a standard marker of nutritional status and overweight in dogs, suggesting probiotic supplementation may support healthier body condition [36]. The Lactobacillus mixture administered to cats showed a reduction in creatinine in cats with chronic kidney disease (CKD), a key indicator of kidney function. These results signify a potential alleviatory effect. Cats with CKD have a high risk of constipation, which leads to unmetabolized amino acids and peptides in the colon. Lactobacillus administration could help improve appetite, activity, and increase the frequency of defecation and moisture content, resulting in softer feces in cats with CKD. This mechanism explains the role of Lactobacillus mixture administration in promoting body waste discharge and reducing its toxicity [39]. While some strains, such as *E. faecium* SF68, showed limited impact on systemic biomarkers [27].

### 3.3. Fecal Quality and Digestibility

Probiotic supplementation has also been associated with enhanced fecal consistency, increased production of short-chain fatty acids, better nutrient digestibility, and improved immune responses in dogs and cats with both normal and sensitive gastrointestinal profiles [6,24,28,29,33]. *Bacillus subtilis* supplementation in dogs reduced fecal *Streptococcus*, *Escherichia coli*, and *Blautia* [38]. Dogs directly fed 62.5 g of microbials, namely *B. subtilis* and *B. licheniformis*, showed improved fecal consistency with less fetid feces compared to dogs on a control diet [40]. Moreover, the concentration of biogenic amines (putrescine, spermidine, and cadaverine) decreased due to different nitrogen substrates that generate catabolites and different utilization rates by gut microbiota [40]. The yeast *S. cerevisiae* var. *boulardii* was utilized as a probiotic in adult cats [41]. *S. cerevisiae* supplementation improved fecal consistency by reducing water content, thereby indirectly supporting overall gut functionality [41]. The IgA levels of the supplemented cats also increased, indicating an anti-infection action on the body’s mucosa and a non-invasive marker of feline intestinal health.

### 3.4. Immune Response and Metabolic Regulation

The probiotic *Weissella cibaria* JW15 (WJW15), isolated from kimchi, could reduce triglycerides in WJW15-fed dogs, indicating a good immune system. Probiotic bacteria can ferment indigestible carbohydrates, produce short-chain fatty acids, and redistribute cholesterol from plasma to lipids, leading to blood lipid reduction [32]. *W. cibaria* JW15 and *B. subtilis* C-3102 demonstrated promising roles in modulating lipid profiles and reducing fecal ammonia [32,33]. *L. johnsonii* CPN23 improved short-chain fatty acid production (acetate and butyrate) and enhanced cell-mediated immune responses in Labrador dogs [28]. These effects highlight the capacity of probiotics to regulate host metabolism and immunity.

Probiotics are live microorganisms that, when consumed in adequate amounts, provide health benefits beyond basic nutrition. In companion animals, supplementation at proper dosage and duration has been shown to modulate gut microbiota, improve fecal quality, and enhance immune function. These findings highlight their strong potential as functional ingredients in pet food, with effectiveness depending on strain specificity, formulation strategy, and host compatibility. Although scientific studies provide evidence for the efficacy of selected probiotic strains, successful commercialization requires technological innovation to ensure viability during processing and storage. This need is reflected in patent activity, where encapsulation technologies and other protective systems are increasingly developed for industrial applications.

**Table 1:** Review of probiotics used in pet food between 2014 to 2025.

Probiotic Strain	Product Process	Factors of Investigation	Major Findings in Pet Health and Function	Pet Testing	Significant Outcome in Food Product Testing	Ref.
Cat						
*S. cerevisiae DSM 34246* (*Canobios-BL*) *var. boulardii*	• Kibble • *Saccharomyces cerevisiae* in powder form	• Control: Kibble without *Saccharomyces cerevisiae* • Treatment: Kibble + *S. cerevisiae* supplementation (5 × 10^9^ CFU/kg of food)	• Decreased BCS indicated good nutritional maintenance. • *S. cerevisiae* supplementation improved gut health, evidenced by reduced fecal score (FS) and humidity (UM), alongside increased fecal dry matter (DM) and IgA (IgA). This indicates sustained good physiological and biological conditions.	• Cats showed good tolerability, safety, and improved fecal consistency with reduced inflammation.		[41]
*L. paracasei*subsp*. paracasei MFM 18* and *L. plantarum*subsp. *plantarum MFM 30-3*	Lactobacillus mixture was blended with chicken and fish oils at 37 °C. One percent was then spread-coated onto commercial pet feed at the same low temperature. Product is commercial pet feed made by Withpet Inc. (Taoyuan, Taiwan)	• CKD cats (2 and 3 stages) were administrated probiotic pet treats daily (10 g) for 8 weeks.	• After 8 weeks of *Lactobacillus* mixture administration, creatinine was reduced or maintained in all cats with chronic kidney disease (CKD). • Gut microbiota shifted significantly from week 0 to week 8, with *Peptostreptococcaceae* decreasing and *Lactobacillaceae* and *Bifidobacterium* increasing.	• Cats with CKD showed improved appetite, activity, and defecation.	• Fecal *L. plantarum* confirmed intestinal survival.	[39]
Dog						
*Lactobacillus acidophilus* GLA09	Supplementation Supplemented feed	• Assays of in vitro tolerance to acid, bile salts; antimicrobial activity; absence of toxic biogenic amines; genomic safety evaluation; vs. in silico controls	• GLA09 exhibits strong gastrointes-tinal tolerance, in-hibits pathogenic bacteria growth, safe (no biogenic amine or transfera-ble virulence risk)		• Genomic and functional traits make it a promising candidate for pet food additive (e.g., heat/acid/bile tol-erance, stress genes, antimicrobial gene clusters)	[6]
*B. subtilis ATCC PTA-122264*	Kibble	• Control: Kibble + maltodextrin placebo • Low: Kibble + 1 × 10^9^ colony-forming units (CFU)/d of *B. subtilis* • High: kibble diet + 5 × 10^9^ CFU/d of *B. subtilis*	• *B. subtilis* supplementation reduced total dry matter, organic matter, and energy digestibility. • Fecal dysbiosis index and the abundance of *Streptococcus*, *Escherichia coli*, and *Blautia* decreased post-supplementation.	• Food energy intake, fecal output, and apparent total tract protein or fat digestibilities remained unchanged.		[42]
*L. plantarum* *CM20-8 (TISTR 2676), L. acidophilus Im10 (TISTR 2734), L. rhamnosus L12-2 (TISTR 2716), L. paracasei KT-5 (TISTR 2688), L. fermentum CM14-8 (TISTR 2720)*	Basal diet: extruded pellets	• Control: Basal diet + maltodextrin as a placebo • Treatment: Basal diet + single probiotic • Treatment: Basal diet + mix probiotic	• Hematology and serum biochemical analysis showed the highest serum creatinine level in the *Lactobacillus fermentum CM14-8* group, while the *L. paracasei KT-5 (TISTR 2688)* group had a lower value than the control. • No significant differences were found in fecal characteristics (ammonia and pH), fecal digestive enzyme activities, serum immunoglobulin (IgG), or fecal IgA between the groups.	• No significant differences were found in body weight, feed intake, body condition score, fecal score, or fecal dry matter across sampling days.		[38]
*B. subtilis* and *B. licheniformis*	Microbials were diluted in poultry viscera oil and put on top of the test diet. Product is basal diet.	• Control (diet without direct feed microbials) Treatment: diet with a microbials 62.5 mg/kg of diet. • *Bacillus subtilis* (3.66 × 10^7^ cfu/kg of the diet) *Bacillus licheniformis* (3.66 × 10^7^ cfu/kg of the diet)	• Microbial supplements reduced fecal protein fermentation and its associated odor and toxicity. • Direct-fed microbials (DFM) supplementation reduced toxic putrefactive/biogenic compounds (putrescine, spermidine, cadaverine, phenols, quinoline) in the intestinal mucosa. • Microbial supplementation increased fecal consistency.		• Diet with microbes had no effect on digestibility.	[40]
*L. kefiri* (LK)	Kefibios^®^ capsule supplement: ≥10^9^ AFUs of viable *L. kefiri* (ISO 19344:2015) per 5 drops in 6 mL vegetable oil	• At T0 (before LK administration), T30 (end of administration), and T60 (one month post-administration)	• At T60, a decreasing trend in *Fusobacteriaceae* and *Ruminococcaceae* was observed. • No significant difference in fecal IgA was found across different time points of LK consumption.		• Quality control revealed KF’s liquid formulation varied from 0.39 × 10^7^ CFU to 7.55 × 10^7^ CFU per five-drop dose.	[34]
*L.acidophilus* D2/CSL (CECT 4529)	Dry commercial diet	• Control: commercial diet • Treatment: Commercial diet + feed additive containing *Lactobacillus acidophilus* (5.0 × 10^10^ CFU g^−1^), premixed with maltodextrins	• *L. acidophilus* improved fecal parameters (fecal score and moisture) in treated dogs. • *L. acidophilus* helped overweight-prone dogs maintain weight and improved fecal parameters.	• Dogs treated with *Lactobacillus acidophilus* showed stable body condition scores (BCS) and thinner skin compared to controls.		[36]
*L. reuteri* AI	Supplementation Supplemented feed	• Fecal microbiome before and after treatment	• *L. reuteri* AI increased or maintained canine *Lactobacillus* sp. levels after 21 days.	• 5 adult dogs, fecal microbiome analysis	• *L. reuteri* AI remained in dog intestines for over a week after feeding. • *L. reuteri* AI exhibits probiotic potential, making it ideal for pet food.	[7]
*L.plantarum* DSM 24730, *S.thermophilus* DSM 24731, *B. breve* DSM 24732, *L.paracasei* DSM 24733, *L.delbrueckii subsp. bulgaricus* DSM 24734, *L.acidophilus* DSM 24735, *B. longum* 120 DSM 24736, and *B. infantis* DSM 24737	Supplementation Vivomixx^®^ multi-strain probiotic powder supplemented feed	• Probiotic mixture and placebo powder (maltose with trace amounts of silicon dioxide) packed in sachets	• Both groups showed a rapid clinical improvement • Probiotic treatment significantly improved clinical recovery by Day 3 (*p* = 0.008), while placebo showed recovery by Day 4 (*p* = 0.002) compared to Day 0. • *Clostridium perfringens* was significantly reduced on Day 7 in the probiotic group (*p* = 0.011), but not in the placebo group.	• 25 dogs with acute hemorrhagic diarrhea syndrome (AHDS), blood, and fecal analysis.	• Dogs with acute hemorrhagic diarrhea syndrome (AHDS): NetF toxin genes rapidly decreased, and clinical recovery was fast in both groups receiving symptomatic treatment, without antibiotics.	[30]
*Weissella. cibaria* JW15 (WJW15)	Supplementation Supplemented feed	• Dietary treatments consisted of basal diet (CON); MJW = CON + 50 g of WJW15 (3.0 × 10^8^ cfu/g); and BJW = CON + 50 g WJW15 (3.0 × 10^9^ cfu/g)	• WJW15, isolated from Korean kimchi, beneficially altered Beagle dog health by reducing serum triglycerides and fecal NH3, while increasing HDL cholesterol and fecal lactic acid bacteria.	• 15 Beagle dogs, fecal and blood parameters	• WJW15 supplementation improved blood lipid parameters in adult Beagles, potentially enhancing their health.	[32]
*B. subtilis* C-3102	Supplementation Supplemented feed	• Control diet and probiotic diet (y 1 × 10^9^ CFU/kg)	• Dogs on the probiotic diet had firmer feces (*p* = 0.011) and higher fecal dry matter during the first two weeks (*p* < 0.05) compared to control dogs. • The probiotic diet reduced NH3 and fecal pH, while increasing short-chain fatty acids (primarily acetate).	• Sixteen adult (aged 4 to 8 years old) Beagle dogs,	• Calsporin^®^ supplementation (1 × 10^9^ CFU/kg) in dog food improved fecal quality, boosted fat and carbohydrate digestion, and supported gut health by lowering ammonia and raising short-chain fatty acids.	[33]
Kefir functional dairy product	Kefir milk preparation: Milk fermented with viable kefir grain	• Gut microbiota was observed on days 0 and 14 of the kefir diet.	• After 14 days of kefir consumption, Fusobacteria (phylum) and the families *Clostridiaceae, Fusobacteriaceae*, and *Ruminococcaceae* significantly decreased. • After 14 days of kefir consumption, *Prevotellaceae*, *Selenomonadaceae*, *Sutterellaceae*, and lactic acid bacteria significantly increased. • Kefir probiotics modified gut microbiota without adverse effects.	• Kefir increased fecal water content, but no body weight loss was observed, indicating no adverse health effects.		[35]
*L. rhamnosus MP01* and *L.plantarum MP02*	• German Sherpherd Junior and Yorkshire Terrier Junior (Royal Canin)	• Control: Commercial food • Treatment: Commercial food + ∼9 log_10_ cfu of *L. rhamnosus MP01* or *L. plantarum MP02*	• Probiotic intake significantly increased fecal *Lactobacillus* and *Faecalibacterium* counts. This also elevated short-chain fatty acid concentrations, indicating reduced gastroenteritis in dogs.		• Both strains showed strong mucin adherence (approximately 12.5% fluorescence retained) but could not degrade gastric mucin in vitro.	[37]
*B. longum* KACC 91563	Coagulation for cheese	• Three groups: Control (no cheese), QC (Queso Blanco cheese without probiotics), and QCB (Queso Blanco cheese with 10^9^ CFU/day of *B. longum* KACC 91563.	• *B. longum* KACC 91,563 (QCB) administration for 8 weeks significantly increased beneficial intestinal bacteria (*Bifidobacterium*, 8.4 ± 0.55) while reducing harmful ones (*Enterobacteriaceae* and *Clostridium*).	• Intestinal microbiota analysis of 15 healthy companion dogs (6 females, 9 males).	• Queso Blanco cheese with B. longum KACC 91,563 positively affected dog gut microbiota and metabolites.	[25]
*B. amyloliquefaciens* CECT 5940	Supplementation Supplemented ration feed	• Control and probiotic-supplemented foods	• Administration of *B. amyloliquefaciens* CECT 5940 increased *Bacillus* count while decreasing coliforms.	• Eight dogs, fecal microbial count	• *B. amyloliquefaciens* CECT 5940 is a promising probiotic with antimicrobial and bactericidal effects for dairy calves and adult dogs.	[26]
*E. faecium* SF68	Supplementation Supplemented ration feed	• Probiotic and control groups	• Probiotic use showed a non-significant rise in mean serum folate at day 14, but a significant drop by day 28 compared to day 1.	• Thirty-six healthy dogs, blood parameters	• No change was observed in canine IBD (inflammatory bowel disease) activity index scores.	[27]
*L. johnsonii* CPN23	Supplementation Supplemented ration feed	• Three groups: CON (no probiotics), dPRO (*L. acidophilus* NCDC 15, dairy-origin), cPRO (*L. johnsonii* CPN23, canine-origin) at 2–3 × 10^8^ CFU per animal/day.	• A 9-week study showed CPN23 supplementation significantly improved (*p* < 0.05) fecal acetate and butyrate, while significantly reducing (*p* < 0.05) fecal ammonia. • Dogs on CPN23 showed a better cell-mediated immune response (*p* < 0.05) compared to CON dogs, as measured by delayed-type hypersensitivity to phytohaemagglutinin-P.	Fifteen adult healthy Labrador female dogs	• No differences in antibody response to sheep erythrocytes were seen among the three groups.	[28]
*L. murinus* (LbP2)	Supplementation	• Probiotic-treated and untreated	• Probiotic-treated dogs showed improved stool, mental status, and appetite.	19 dogs (>60 days old) with distemper and diarrhea: clinical signs included stool output and consistency, vomiting, appetite, and mental status.	• Probiotics show promise for canine distemper diarrhea.	[29]
*L. plantarum* CIDCA 83114	Baking (biscuit 140 °C for 45 min and coating at 30 °C for 40 min)	• Biscuit ingredients: Wheat flour and wheat flour + soy flour • Coating: starch coating and starch + inulin coating	• Coating significantly increased the viability of *L. plantarum* on wheat biscuits after simulated gastrointestinal passage, with the starch + inulin coating yielding the best results. • Inulin in the coating did not significantly affect *L. plantarum* viability in wheat + soy flour biscuits	-	• Coated biscuits retained *L. plantarum* counts above 10^8^ CFU/mL after one month of storage. • Inulin at 20 g/L did not significantly affect bacterial survival after one month.	[9]
*B. amyloliquefaciens* CECT 5940 and *Enterococcus faecium* CECT 4515	Supplementation Supplemented feed	• Control and probiotic treatment (1 × 10^8^ CFU)	• During supplementation, pathogenic Clostridia significantly dropped in the probiotic group (2.94 ± 0.53 CFU/g feces vs. 5.64 pre-supplementation; *p* < 0.001).	• Sixteen beagle dogs: Microbial enumeration and pH measured pre-supplementation, post-supplementation, and after 6 days of withdrawal.	• Fecal scores and digestibility coefficients did not differ between control and probiotic groups. • No statistical differences were found in most microbiota or fecal pH.	[31]
*L.acidophilus* DSM 13241	Extrusion and post-extrusion coating for dry dog food	• Control and probiotic diets	• Probiotic feeding improved fecal consistency, dry matter, and defecation frequency.	Defecation frequency, fecal quality, and nutrient digestibility in six adult German Shorthair Pointers.	• *L. acidophilus* DSM 13,241 stabilizes canine digestion.	[24]
*L. acidophilus strain* DSM13241	Extrusion for Kibble followed by coating (probiotic in an oil matrix).	• Addition and without addition of *L. acidophilus*	• The probiotic bacterium was detected in feces by ribotyping and RNA gene sequencing during administration, but not two weeks after cessation. • Probiotic food intake increased fecal lactobacilli and decreased clostridial organisms. • RBCs, Hct, hemoglobin, neutrophils, monocytes, and serum immunoglobulin G significantly increased, while RBC fragility and serum NO decreased.	15 adult dogs, fecal and blood parameters	• *L. acidophilus* survival in the supplemented food was 71% initially and 63% at the study’s end, demonstrating bacterial viability during manufacture and storage.	[2]

## 4. Patent of Probiotics Used in Pet Food

The field of pet nutrition has increasingly recognized the importance of the gut microbiome in overall animal health as shown in Table 2. Consequently, the incorporation of probiotics (live microorganisms that, when administered in adequate amounts, confer a health benefit on the host) has become a significant area of innovation in pet food formulations. This has naturally led to considerable interest and activity in patenting novel probiotic strains, delivery methods, and pet food compositions designed to enhance the well-being of our companion animals. Our discussion will delve into the landscape of patents related to probiotics used in pet food, exploring examples of patented technologies and the types of microorganisms commonly employed to promote digestive health, immunity, and other beneficial effects in pets.

### 4.1. Digestive Health and Fecal Quality

Chen et al. (2023) [43] describe a specialized pet food product for cats and dogs: low-temperature baked active probiotic granules. The formulation features a meat-rich base and functional ingredients, including *B. licheniformis*, *B. subtilis*, and *C. butyricum*. The controlled baking process (55–60 °C) preserves nutrient bioactivity and probiotic viability while enhancing palatability and safety. The product also incorporates prebiotics, omega-rich oils, and botanical extracts to support gut health and immune balance. Jin et al. (2021) [44] present a scientifically formulated dry cat food designed to enhance digestive health and nutrient absorption. This is achieved through postbiotic microcapsules containing *B. licheniformis and B. subtilis*, protected by a dual-layer alginate-chitosan shell for controlled intestinal release. The formulation also includes β-glucans, enzymes, and prebiotics, with enzymatically hydrolyzed meat-based ingredients to improve peptide absorption. Clinical tests demonstrated significant improvements in stool consistency, digestibility, and intestinal flora balance in cats. Liu et al. (2021) [45] detail a puffed dry pet food incorporating a multi-strain probiotic mix (*L. acidophilus, B. longum, B.subtilis*, and *L. plantarum*) designed to enhance digestion and nutrient absorption. The food utilizes enzymatically hydrolyzed meat, is processed via extrusion puffing and oil spray-coating, and has probiotics added post-cooling to ensure viability. Canine trials showed improved stool quality, increased food intake, and shinier coats, highlighting the benefits of probiotic synergy and careful processing for functional pet foods.

### 4.2. Stability and Processing Innovations

Li et al. (2022) [46] introduce a dry pet food with visible embedded food materials containing functional particles. These particles are fortified with thermosensitive bioactives (*B.acillus subtilis*, *B. coagulans*, lactic acid bacteria, and yeast) coated using fluidized-bed technology, and then incorporated into the kibble. This method protects these sensitive ingredients from heat degradation during manufacturing, ensures delayed release in the digestive system, and enhances the product’s visual appeal, nutrient retention, and digestive health benefits through a novel synbiotic delivery system. Chinachoti et al. (2020) [47] present a pet food formulated with viable, shelf-stable probiotics, including *B. coagulans* GBI-30, 6086 and *B. subtilis*, added via low-temperature post-processing techniques such as microencapsulation and dry mixing. These heat-tolerant probiotics are selected to ensure survival and provide digestive and immune health benefits in pets, offering a stable and palatable functional food option that meets industrial needs without requiring cold storage.

### 4.3. Immune Modulation and Stress Resistance

Wang et al. (2024) [48] introduce a functional dry cat food designed to regulate stomach function and support gastrointestinal balance through a synergistic blend of *E. faecium*, *S. lactis*, *S. cerevisiae*, and *B.subtilis*, along with oligosaccharide prebiotics. The food is produced using steam cooking, enzymatic fractionation, extrusion, vacuum-layered spray-coating, and low-temperature baking to maintain nutrient quality and palatability. This results in a highly digestible food that improves microbial balance, reduces gastric sensitivity, and supports optimal feed intake, demonstrating benefits for stomach health and microbiota stability. Jiang et al. (2021) [49] detail a nutritionally complete pet food enhanced with immune-boosting agents, including *B. longum* and *L. casei*. The formula incorporates proteins, vitamins, polysaccharides, and polyunsaturated fatty acids from plant, animal, and microbial origins, processed using various methods with post-processing addition of immune enhancers. Clinical trials showed a 15% reduction in pet infectious diseases, underscoring the immunomodulatory benefits of this formulation and the trend toward therapeutic pet nutrition. Liu et al. (2021) [50] disclose a pet food formulation enriched with antioxidants and a probiotic complex (*L. acidophilus, B. longum, B. subtilis, L. plantarum*). The manufacturing process involves extrusion and drying, with a post-processing spray of probiotics and nutritional oils to ensure viability. This product aims to enhance immunity, stress resistance, and gut health, with hydrolyzed chicken meat further supporting nutrient absorption and gastrointestinal function, showing benefits in canine stress trials.

### 4.4. Postbiotics and Paraprobiotics

Brashears et al. (2020) [51] outline a multiple inhibition system employing lactic acid bacteria (LAB), including *L. salivarius* strains L14 and L28 and *E. hirae*, to significantly reduce *Salmonella*, *Listeria monocytogenes*, and *E. coli* in pet foods. The LAB are applied through probiotic-enriched chicken fat coatings on kibble or directly incorporated into various feed types, achieving up to a 7-log reduction in *Salmonella* within 72 h and complete inhibition in some cases. These probiotics are also safe, palatable, and easily integrated into current production methods, offering a non-chemical approach to enhance pet food safety and support microbial balance. Berger et al. (2014) [52] introduce an innovative pet food composition utilizing non-viable probiotics, specifically *Lactobacillus farciminis*, which maintain health-promoting effects despite heat inactivation. This technology allows for the incorporation of these paraprobiotics into dry pet food formats without thermal degradation concerns, providing benefits such as improved gut health and immune modulation, while also simplifying storage and extending shelf life.

### 4.5. Next-Generation Delivery Systems

Yan (2021) [53] introduces a functional pet snack formulated to prevent obesity through a low-calorie and high-protein composition. This snack includes a blend of probiotics (*C. butyricum, Pediococcus, B. subtilis, B. licheniformis*, and lactic acid bacteria) combined with nutrient-rich ingredients such as chicken breast, egg whites, and seaweed-extracted oligosaccharides. Designed to enhance gastrointestinal motility and nutrient absorption, promoting gut health and satiety while minimizing caloric intake, the treat is processed via cooking, blending, extrusion, and low-temperature dehydration to support digestive efficiency and weight management. Zhuang and He. (2022) [54] discloses an intelligent PBM (Probiotic, Balanced, Meal) feeding box for pets that automates the dosing and mixing of live probiotics (*B. coagulans, L. plantarum, B. animalis*, and *S. boulardii*), inactivated bacterial liquids, and freeze-dried food materials from separate compartments. Utilizing microencapsulation for probiotic stability and integrated heating and water systems for optimized rehydration and sanitation, this feeding box aims to address food spoilage, dosing inaccuracies, and gut health by providing fresh, nutrient-rich meals that enhance gut microbiota, reduce fecal odor by over 90%, and improve gastrointestinal immunity in pets. Bai (2020) [55] presents a pet meal box system comprising separately packaged dry kibble, fresh-cooked ingredients, and a nutritional supplement containing probiotics (*B. subtilis* and *C. butyricum*), enzymes, prebiotics, and essential vitamins. This design aims to preserve freshness and functional integrity, enabling pet owners to create balanced, mixed meals with enhanced digestibility and immune function, compensating for nutrient losses during cooking or processing.

Dry food remains the dominant format for probiotic pet food, reflecting consumer preference for convenient, long-shelf-life products and the market’s focus on kibble formulations [1]. However, extrusion and drying—key processes in dry food manufacturing—expose probiotics to high temperature, pressure, and dehydration stress, resulting in significant reductions in viability [7,8,9]. This technical bottleneck explains why, despite strong consumer demand for probiotic kibble, scientific studies often report inconsistent probiotic counts at the point of consumption. To address this challenge, patented technical solutions have emerged, including microencapsulation technologies that shield probiotics within protective matrices [52,56], low-temperature baking processes that minimize thermal inactivation [45], and hydrogel-based drying systems that preserve viability during storage and gastrointestinal transit [56].

Despite growing patent portfolios and commercial launches, several bottlenecks remain unresolved. The next section therefore highlights challenges in maintaining probiotic viability, harmonizing regulatory frameworks, and translating research into consistent industrial practice.

**Table 2:** Invention patent of probiotics used in pet food.

Probiotic Strain	Food Process	Product	Major Claim	Patent Type	Country of Patent Applicants	Patent Name	Ref.
*E. faecium*, *S. lactis*, *S. cerevisiae*, *B.subtilis* (*with prebiotics*)	Steam cooking, enzymatic separation, mixing, extrusion, vacuum spray coating, low-temp baking	Stomach-conditioning functional cat food	Enhanced digestive health, improved gut flora balance, high palatability and nutrient retention	Invention patent	China	Preparation method of cat food with prebiotic stomach conditioning effect (119423228A)	[48]
*B. licheniformis, B. subtilis, C. butyricum*	Low-temperature baking (55–60 °C), kneading, precision mixing, extrusion molding	Active probiotic nutrition granules	Enhanced palatability, stable probiotic activity, nutrient integrity	Invention patent	China	Low-temperature Gao Rou baked active probiotic nutrition granule for dogs and cats and preparation method (116172134A)	[43]
*L. casei* *B. longum*	Pelletizing and coating with functional ingredients	Immune-enhancing pet food	Immune modulation and infection reduction	Invention patent	China	Complete pet food with immune enhancement function (113951396A)	[49]
*B. licheniformis B. subtilis*	Low-temp baked granulation	Soft baked granules	Maintained activity under low heat; balanced nutrition	Invention patent	China	Low-temperature baked active probiotic nutrition granule (113925114A)	[44]
*B. subtilis, B. coagulans, Lactic acid bacteria, Yeast*	Embedding into fortified particles, extrusion-puffing, fluidized-bed coating	Granular staple pet food with visible embedded materials	Improved nutrient retention during processing, controlled release in the gut, clear visibility of functional food inclusions	Invention patent	China	Granular staple food pet food embedded with food materials (114868837A)	[46]
*B. coagulans, L. plantarum, B. animalis, E. faecium, S. thermophilus, S. boulardii, Photosynthetic bacteria (varied combinations)*	Separate storage and metered delivery of live powders and inactivated liquids, microencapsulation for freeze-dried inclusion, automatic water and food mixing	PBM probiotic automated meal box	Precision feeding of viable and inactivated probiotics; improved gut flora, reduced fecal odor, and increased gut immunity	Design patent	China	Pet PBM probiotics feeding box (216874426U)	[54]
*C. butyricum, Pediococcus, B. subtilis, B. licheniformis, Lactic acid bacteria*	Cooking, ingredient blending, probiotic mixing, pellet extrusion, low-temp dehydration	Anti-obesity health snack for pets	Low-calorie, high-protein formulation promoting gut peristalsis and nutrient absorption	Invention patent	China	Health food for preventing pet from getting fat (114304382A)	[53]
*Bifidobacterium, Bacteroides, Clostridium, Fusobacterium, Melissococcus, Propionibacterium, Streptococcus, Enterococcus, Lactococcus, Staphylococcus, Peptostrepococcus, Bacillus, Pediococcus, Micrococcus, Leuconostoc, Weissella, Aerococcus, Oenococcus, or Lactobaccillus.*	Low-temperature addition post-extrusion, microencapsulation, shelf-stable inclusion	Functional pet kibble and treats with viable probiotics	Shelf-stable, heat-tolerant probiotics added post-processing to improve gut health	Invention patent	United States	Pet food compositions including probiotics and methods of manufacture and use thereof (11510424B2)	[47]
*L. plantarum* and *B. subtilis*	Extrusion + probiotic spraying post-drying	Complete antioxidant-rich pet food	Improved immunity and stress resistance	Invention patent	China	Antioxidant-containing pet complete food and preparation method (112913981A)	[50]
*L. acidophilus, B. longum, B. subtilis, L. plantarum*	Enzymatic hydrolysis, extrusion puffing, oil spray-coating, probiotic mixing post-cooling	Puffed dry pet food	Puffed dry pet food	Invention patent	China	Puffed pet food and preparation method thereof (112450319A)	[45]
*Saccharomyces cerevisiae, B. subtilis, C. butyricum*	Separate packaging, mixing probiotics into nutrient supplements with prebiotic carriers	Meal box pet food (mixed rice + supplements)	Nutritional balance and probiotic support through combined fresh and dry ingredients	Invention patent	China	Pet meal box mixed rice (111053162A)	[55]
*L. salivarius (L14, L28), E. hirae (L14)*	Coating on pet kibble using probiotic-enriched chicken fat; direct mixing or spraying	Pathogen-inhibiting dry kibble and treats	Reduction in Salmonella and other pathogens in pet food; improved food safety	Invention patent	United States	Reduction in Pathogens and Other Bacteria in Food and Feed Products Utilizing a Multiple Inhibition System with Lactic Acid Bacteria (20200288750A1)	[51]
Heat-inactivated *Lactobacillus farciminis*	Non-viable inclusion, spray or mix	Dry pet food containing *L. farciminis* (non-viable form)	Gut health benefits with shelf-stable inactive probiotics	Invention patent	International	Probiotic pet food composition with non-viable probiotics (2015004055A1)	[52]
*L. paracasei*	Vacuum Drying, Hydrogel Matrix	Crunchy Flakes, Treats	Long-term probiotic viability, gastric survival	Invention patent	Japan	Dry food products containing live probiotics (5285617B2)	[56]
*E. faecium* NCIMB 10415	Encapsulation Drying	Functional kibble	Improved fecal consistency and GI health	Invention patent	United States, UK, France	A method for the management of fecal consistency in dogs (2010122104A1)	[57]

## 5. Factors Affecting the Viability of Probiotics During Processing and Storage

The viability of probiotics is a critical factor in their ability to deliver various health benefits [58]. However, probiotic viability depends on several factors. These factors include carrier intrinsic factors such as pH, oxygen, water activity, and other components (sugar, salt, hydrogen peroxide, bacteriocin, etc.), processing parameters including heat treatment and storage conditions, and finally, microbiological parameters (strain of probiotics employed, rate, and proportion of inoculation) [59].

Criteria such as suitability in terms of safety, stability, and compatibility between the probiotic strains and the film matrix need to be considered. The stability and viability of probiotics are influenced by pH, redox potential, and surface tension of the film-forming solution. It is essential to choose probiotic strains with proven health benefits and a high level of viability and stability during processing and storage to maintain their functionality in the final product. The selected probiotic strains should be compatible with the additives used in the film formulation, such as plasticizers and surfactants, which are essential for the mechanical properties and functionality of the edible films [60,61,62].

### 5.1. Chemical Factors

The presence of oxygen can affect the viability of probiotics during processing and storage. The effects of oxygen on probiotic viability vary widely depending on the genera. Most lactobacilli are microaerophilic while bifidobacteria are identified as anaerobic with a high level of sensitivity to oxygen. Therefore, the oxygen concentration and oxygen permeability of the packaging must be set at a low level to effectively control the loss of probiotic viability. The sensitivity of anaerobic bacteria to oxygen limits their survival and use in industrial applications [63,64]. In contrast, facultative anaerobes like *B. coagulans* exhibit greater oxygen tolerance. These bacteria can consume oxygen within the gastrointestinal tract, reducing oxidative stress and creating a more favorable environment for anaerobic microorganisms such as *Lactobacillus* and *Bifidobacterium* [65].

Water activity (a_w_) is another critical factor influencing probiotic survival. Elevated water activity can induce a state of partial cell activity, where certain enzyme systems remain functional while others are suppressed. This imbalance can lead to the intracellular accumulation of metabolic intermediates, ultimately impairing microbial viability [66]. The a_w_ plays an important role during storage [67]. Vesterlund et al. (2012) [68] demonstrated a correlation between higher a_w_ and accelerated probiotic decline in dry foods stored for 14 months. Maintaining a low a_w_ environment can significantly extend the shelf life of dry probiotic products.

The addition of ingredients can significantly impact probiotic viability in fermented and non-fermented products. These effects can be protective, neutral, or detrimental to probiotic stability, making ingredient compatibility crucial for probiotic survival. Common additional ingredients include salt (KCl and NaCl), sugar (lactose and sucrose), sweeteners, aroma compounds, natural and synthetic colorings, flavor agents, nisin, natamycin, lysozyme, and nitrates [59,69]. Addition of sugars, vitamins, minerals and prebiotics in probiotic products may promote the growth of probiotics. Skim milk powder, whey protein and lactose possibly act as protectants against processing conditions. Carrier matrix may also influence the survivability of probiotics. Novel approaches using edible film and coatings have been shown to improve probiotic cell survivability [64]. Carbohydrates, protein and fat may act as protective agents to probiotic cells. Carbohydrates, such as lactose, can be used as a drying matrix, acting as a substitute for the hydrogen-bonded water in the head of a group of phospholipid bilayers in the probiotic cell membrane. Furthermore, if carbohydrates form a glassy state during drying, the resulting high viscosity can act as a protective encapsulation for probiotics, limiting water and oxygen exchange [4,5,6,7,8,9,10,11,12,13,14,15,16,17,18,19,20,21,22,23,24,25,26,27,28,29,30,31,32,33,34,35,36,37,37,38,39,40,41,42,43,44,45,46,47,48,49,50,51,52,53,54,55,56,57,58,59,60,61,62,63,64,65,66,67,68,69,70,71]. Prebiotics, such as inulin, polydextrose, wheat dextrin, fructooligosaccharides (FOS), and galactooligosaccharides (GOS), are increasingly used to enhance probiotic viability during processing and storage. These prebiotics are particularly valuable when relatively invasive techniques like spray-drying or freeze-drying are employed [72]. Maltodextrin has been reported to have benefits as a prebiotic that stimulates probiotic growth. It also functions as a protectant for bacteria at high temperatures and pressures, therefore increasing probiotic survival during the drying process [73]. Resistant-to-digestion maltodextrin (RMD) is a non-viscous soluble fiber created by modifying alpha-1,4-glucose linkages to random 1,2-, 1,3-, and 1,4-alpha or -beta linkages, which resist digestion. Being resistant to digestion, RMD is considered as prebiotic, fermented by intestinal bacteria and has been shown to positively impact gastrointestinal homeostasis [74]. Lieu et al., (2020) [75] reported the addition of maltodextrin has shown a significant increase in protection capacity on the viability of *L. plantarum* during the baking process of cupcake. Anekella and Orsat (2013) [76] microencapsulated probiotics using maltodextrin via spray-drying. They found that a sub-lethal heat pre-treatment allowed the cells to survive within a certain temperature range but were destroyed at temperatures exceeding 92 °C.

### 5.2. Physical Factors

Processing significantly impacts probiotic viability. Heat treatment, dehydration, and mechanical stress can disrupt cell integrity and osmotic balance, leading to reduced cell viability. Optimizing processing conditions tailored to the probiotic strain can increase its viability [77,78]. Mechanical stress can negatively impact the viability and functionality of probiotics. Mixing process contributing to disruption of probiotic membrane cell and cell injuries interfering functionality and metabolic activities [79,80].

Dehydration subjected probiotic cells to significant stress. Intracellular water removal exerts mechanical pressure on the cell membrane, altering its plasticity. Along with desiccation stress, cells have to face several specific stresses, which may lead to severe loss in viability, unless protected using protectant and developed with specific efficient protocols [81].

Probiotic cells are highly susceptible to thermal inactivation. Excessive temperatures can lead to the degradation of biomolecules like proteins and nucleic acids, impairing their function and inhibiting cellular metabolism. High temperatures can also disrupt cellular activities by increasing membrane fluidity. Temperatures exceeding 45 °C can be detrimental to most probiotics. Pasteurization processes, typically conducted at 60 °C, can significantly reduce probiotic viability by up to 3 log CFU. The tolerance of individual probiotic strains to heat varies widely, with psychrophilic and mesophilic strains generally exhibiting lower thermal stability compared to thermophilic strains such as *Bacillus* species [58,82]. Storage temperature significantly impacts probiotic viability. The higher the storage temperature, the lower the probiotic survivability over time. Refrigerated temperature storage generally serves the best probiotic stability [67].

### 5.3. Microbiological Factors

Probiotic strain type significantly influences how various factors impact viability. Different species exhibit markedly different tolerances to various conditions. Probiotic inoculum level can significantly influence viability during fermentation and cold storage. While a sufficient initial inoculum is essential for achieving therapeutic effects upon consumption, excessive inoculation may lead to nutrient deficiencies during fermentation and storage, potentially compromising probiotic survival [63]. Iaconelli et al., (2015) [83] reported *L. plantarum* least affective by the drying process compared to *Bifidobacterium bifidum* that highly sensitive to drying process. Salimiraad et al. (2022) [84] in their study showed that *B. coagulans* significantly exhibited a greater survival rate (6.35 log CFU/g) compared to *L. casei* (6.01 log CFU/g) during storage for 14 days at 4 °C.

Lactobacillus, Bifidobacterium, Saccharomyces, Enterococcus, Streptococcus, Pediococcus, Leuconostoc, and Bacillus are probiotic genera that mainly provide health benefits, with Lactobacillus and Bifidobacterium being the strains most used in foods containing probiotics. However, most of these strains cannot tolerate harsh conditions due to non-sporulation characteristics that adversely affect their viability. Among many genera, Bacillus is noted for its ability to form spores, which are highly resistant to technological stresses and extreme conditions, such as low pH, and remain stable across a wide range of temperatures. Bacillus, as a probiotic, can form spores both in aerobic and facultatively aerobic conditions [85,86,87].

## 6. Challenges and Future Opportunities

**Figure 4:**
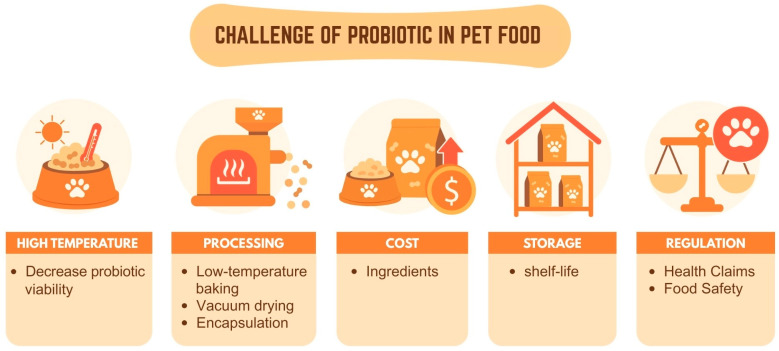
Challenge of probiotic in pet food.

Despite burgeoning interest, integrating probiotics into pet food faces significant hurdles, as shown in Figure 4. A primary challenge is probiotic viability during manufacturing, storage, and passage through a pet’s harsh gastrointestinal tract. High temperatures and pressures during extrusion, oxygen exposure, and long shelf lives can severely reduce live bacterial counts, compromising efficacy. Strain specificity is another concern; not all probiotic strains benefit all companion animal species or health conditions. Research often shows contradictory results, highlighting the need for more species-specific and targeted studies (e.g., canine versus feline gut microbiomes differ) [88]. Furthermore, regulatory frameworks are still evolving, requiring robust scientific evidence for health claims and consistent quality control to ensure product safety and efficacy. The cost of premium ingredients, palatability issues, and owner skepticism about the necessity of supplements also present market barriers. Moreover, the selection of probiotic strains is directly associated with technological strategies that preserve their viability under industrial conditions. For example, spore-forming Bacillus spp. are naturally suited for high-temperature extrusion, while non-spore-forming Lactobacillus spp. often require protective systems such as microencapsulation. These delivery technologies not only ensure survival through processing and storage but also enable the realization of health claims, including improved gastrointestinal function and immune regulation, as demonstrated in controlled trials. Thus, effective probiotic pet food development requires a combined evaluation of strain resilience, technological feasibility, and scientifically validated health outcomes.

Probiotic pet food must adhere to several key requirements to ensure safety, efficacy, and consumer trust. Strains must be precisely identified at the genus, species, and strain level (e.g., Lactobacillus *acidophilus DSM13241*) [2,30], with GRAS or QPS status [13] and documented evidence of safety and efficacy in dogs and cats [30]. Viable counts should be guaranteed until the end of shelf life, typically ≥10^6^–10^9^ CFU/g [32,33,34,70], with dosages aligned to evidence-based benefits [34]. Probiotics should not negatively affect palatability, aroma, or texture, [43,48] and only strains resilient to processing (e.g., Bacillus *coagulans*, *B. subtilis*) [19,85,86,87] or those protected by microencapsulation or post-extrusion coating should be used to maintain stability [2,9,24]. Storage and transportation require controlled temperatures (preferably <25 °C [38,59], refrigeration for sensitive strains), oxygen- and moisture-barrier packaging [38], and protection against humidity or temperature fluctuations [42]. Finally, labeling must clearly state the genus, species, and strain, guaranteed CFU counts at the end of shelf life, feeding recommendations, target species, and storage instructions, with all health claims substantiated by scientific evidence.

The future of probiotics in pet food is bright, driven by increasing pet humanization and owner awareness of gut health. Advanced encapsulation technologies offer solutions to improve probiotic survival, ensuring more live bacteria reach the intestine [89]. The development of postbiotics and synbiotics (probiotics combined with prebiotics) presents novel formulations that can offer benefits even if live bacteria do not survive or synergistically enhance their effects. Research is moving towards identifying and utilizing host-derived, species-specific strains that are naturally adapted to the pet’s gut.

While this review aims to summarize current knowledge on probiotics in pet food, several limitations should be acknowledged. First, published research specifically targeting dogs and cats remains limited compared to studies in humans and livestock, making it necessary to extrapolate some findings. Second, many commercial formulations contain proprietary blends, and detailed strain-level information or dosage data are often not publicly available, restricting transparency in comparing products. Third, variability in study design, animal models, and probiotic strains used across the literature makes direct comparison challenging. Finally, regulatory frameworks and labeling standards for probiotic pet food vary across regions and are still evolving, which may limit the generalizability of some recommendations provided in this review.

## 7. Conclusions

The pet food industry has demonstrated substantial innovation, particularly in the selection of resilient probiotic strains such as *L. acidophilus*, *B. longum*, *B. subtilis*, and *S. cerevisiae*, and in the development of advanced delivery technologies like microencapsulation, post-extrusion coating, and hydrogel matrices to preserve probiotic viability during processing and storage. Research evidence consistently supports the ability of probiotics to positively modulate gut microbiota, improve fecal quality, enhance short-chain fatty acid production, and strengthen mucosal immunity in dogs and cats. However, challenges persist regarding the maintenance of probiotic stability throughout shelf life, strain-specific health outcomes, and the development of targeted formulations for different life stages and health conditions. Furthermore, although product launches and patent filings have surged, academic research on strain efficacy, dose optimization, and long-term health impacts remains comparatively limited, highlighting an important gap for future studies. The continued success of probiotic pet foods will depend on interdisciplinary advancements across microbiology, food processing, veterinary science, and nutrition. Future research should focus on validating strain-specific benefits through controlled clinical trials, exploring the synergistic effects of combining probiotics with prebiotics or postbiotics, and designing tailored solutions that address the diverse physiological needs of companion animals. Overall, probiotics represent a promising avenue for enhancing the quality, safety, and functionality of pet foods, supporting the growing demand for health-promoting dietary strategies in the pet nutrition sector.

## Data Availability

The original contributions presented in the study are included in the article, further inquiries can be directed to the corresponding author.
